# Supplementary material for: Identification of photoperiod-induced specific miRNAs in the adrenal glands of Sunite sheep (Ovis aries)
Source: Front Vet Sci. 2022 Jul 22;9:888207. doi: 10.3389/fvets.2022.888207 (PMC9354845; doi:10.3389/fvets.2022.888207)
Supplement: Supplementary file 1 [file Data_Sheet_1.ZIP › Supplementary file/miRDeep2 file.zip!/miRDeep2 file/Novel_Structure/SPLP42A2/Novel_638.pdf]

| | | |
|---|---|---|
| Provisional ID | : | NC_019470.2_26761 |
| Score total | : | 2 |
| Score for star read(s) | : | -1.3 |
| Score for read counts | : | 0 |
| Score for mfe | : | 1.7 |
| Score for randfold | : | 1.6 |
| Score for cons. seed | : | |
| Total read count | : | 26 |
| Mature read count | : | 21 |
| Loop read count | : | 0 |
| Star read count | : | 5 |

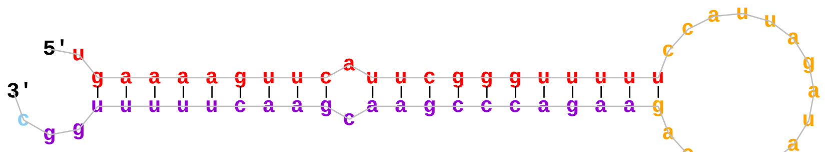


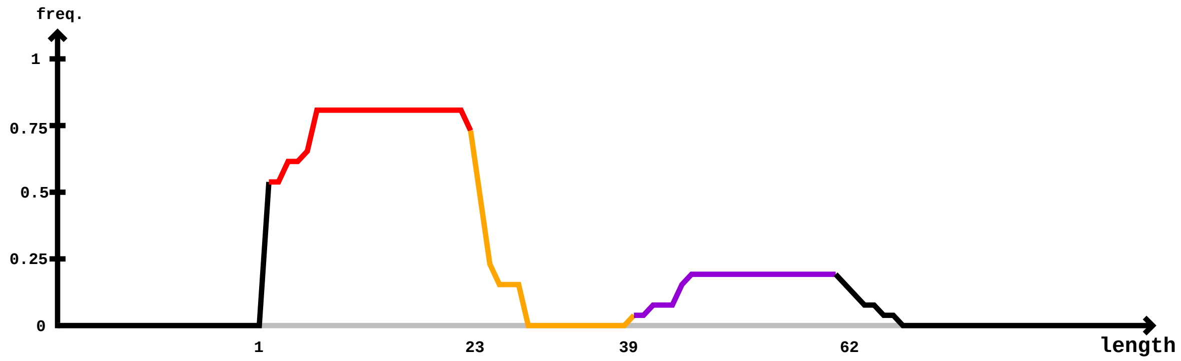


**Mature** **Star**

```
5'- cugccuucuauugggcuggcugaaaaguucauucggguuuuuccauuagauaguacagaagacccgaacgaacuuuuuggccaaccaauacauuuuccaucucuguucuucc   -3'    obs
    cugccuucuauugggcuggcugaaaaguucauucggguuuuuccauuagauaguacagaagacccgaacgaacuuuuuggccaaccaauacauuuuccaucucuguucuucc          exp
    ........((((((..((((((((((((((.(((((((((((...............))))))))))).)))))))))))))).))))))......................   reads   mm   sample
    ....................ugaaaaguucauucggguuuu.......................................................................   2       0    seq
    ....................ugaaaaguucauucggguuuuu......................................................................   12      0    seq
    ......................aaaGguucauucggguuuuuc.....................................................................   1       1    seq
    ......................aaaaguucauuAggguuuuucc....................................................................   1       1    seq
    ........................aaguucauucggguuuuucc....................................................................   1       0    seq
    .........................aguucauucggguuuuuccauu.................................................................   4       0    seq
    ..........................................................aagacccgaacgaacuuuuugg................................   1       0    seq
    ............................................................gacccgaacgaacuuuuuggc...............................   1       0    seq
    ...............................................................ccgaacgaacuuuuuggccaa............................   1       0    seq
    ...............................................................ccgaacgaacuuuuuggccaacU..........................   1       1    seq
    ................................................................cgaacgaacuuuuuggcc..............................   1       0    seq
```
